# Host size influences the effects of four isolates of an amphibian chytrid fungus

**DOI:** 10.1002/ece3.3255

**Published:** 2017-10-03

**Authors:** Angela K. Burrow, Samantha L. Rumschlag, Michelle D. Boone

**Affiliations:** ^1^ Department of Biology Miami University Oxford OH USA

**Keywords:** *Batrachochytrium dendrobatidis*, chytridiomycosis, host condition, host size, host–pathogen interactions, multiple stressors

## Abstract

Understanding factors that influence host–pathogen interactions is key to predicting outbreaks in natural systems experiencing environmental change. Many amphibian population declines have been attributed to an amphibian chytrid fungus, *Batrachochytrium dendrobatidis* (*Bd*). While this fungus is widespread, not all *Bd‐*positive populations have been associated with declines, which could be attributed to differences in pathogen virulence or host susceptibility. In a laboratory experiment, we examined the effects of *Bd* isolate origin, two from areas with *Bd*‐associated amphibian population declines (El Copé, Panama, and California, USA) and two from areas without *Bd*‐related population declines (Ohio and Maine, USA), on the terrestrial growth and survival of American toad (*Anaxyrus americanus*) metamorphs reared in larval environments with low or high intraspecific density. We predicted that (1) *Bd* isolates from areas experiencing declines would have greater negative effects than *Bd* isolates from areas without declines, and (2) across all isolates, growth and survival of smaller toads from high‐density larval conditions would be reduced by *Bd* exposure compared to larger toads from low‐density larval conditions. Our results showed that terrestrial survival was reduced for smaller toads exposed to *Bd* with variation in the response to different isolates, suggesting that smaller size increased susceptibility to *Bd*. Toads exposed to *Bd* gained less mass, which varied by isolate. *Bd* isolates from areas with population declines, however, did not have more negative effects than isolates from areas without recorded declines. Most strikingly, our study supports that host condition, measured by size, can be indicative of the negative effects of *Bd* exposure. Further, *Bd* isolates’ impact may vary in ways not predictable from place of origin or occurrence of disease‐related population declines. This research suggests that amphibian populations outside of areas experiencing *Bd*‐associated declines could be impacted by this pathogen and that the size of individuals could influence the magnitude of *Bd*'s impact.

## 1 INTRODUCTION

While species extinctions and extirpations have long been attributed to habitat loss, climate change, invasive species, and overharvesting (Pimm, Russell, Gittleman, & Brooks, 1995; Wilcove, Rothstein, Dubow, Phillips, & Losos, 1998), only lately have we considered infectious diseases, which can interact with these other threats, as a force that can shape biodiversity (Smith, Sax, & Lafferty, 2006; Wilcove et al., 1998). Recently, rates and severity of infectious disease outbreaks have increased (Daszak, Cunningham, & Hyatt, 2000; Jones et al., 2008; Smith, Acevedo‐Whitehouse, & Pedersen, 2009) across taxa (Daszak et al., 1999; Martel et al., 2013; McCallum & Jones, 2006), prompting an urgent need to explore the effects that pathogens can have on biodiversity by evaluating the responses of hosts to pathogen across varying environmental conditions.

The outcome of the introduction of a pathogen into a naïve host population is difficult to predict because of complex interactions among the host, the pathogen, and the environment in which they exist. For instance, host susceptibility and pathogen virulence can vary across environmental conditions, determining the outcome of pathogen exposures on hosts (Daszak et al., 2000; Smith et al., 2006). Additionally, genetic variation in hosts and pathogens can also influence the outcome of host–pathogen interactions (Thrall, 2003). Outbreaks of infectious diseases can be caused by unintentional pairings of novel strains of pathogens with naïve host populations via commercial trade or host relocations and reintroductions (Cunningham, 1996; Daszak et al., 2000; Mathews, Moro, Strachan, Gelling, & Buller, 2006). Understanding variation in hosts’ responses to different strains of pathogens across environmental conditions will become essential as the world becomes more connected through global commerce and trade, conservation efforts to relocate organisms, and climate change‐induced shifts in distributions of hosts and pathogens. If our goals are to target host populations at risk to novel pathogen exposure, predict infectious disease outbreaks, and manage host populations to limit risk, understanding how variation in hosts and pathogens can mediate disease development will improve our success of these efforts. For instance, by incorporating variation in host responses across strains of pathogens into predictive models and management plans, we can improve the efficient use of management resources and limit exposure of hosts to unnecessary risks (Gaydos et al., 2002; Goldberg, Coleman, Grant, Inendino, & Philipp, 2003; Yates, Antia, & Regoes, 2006).

Amphibians have been experiencing global declines that, in part, are linked to infectious disease (Barinaga, 1990; Stuart et al., 2004). Many declines are associated with a chytrid fungal pathogen, *Batrachochytrium dendrobatidis* (hereafter, *Bd*), which causes the disease chytridiomycosis (Berger et al., 1998; Daszak, Cunningham, & Hyatt, 2003; Lips et al., 2006). *Bd* is broadly distributed worldwide, which has been aided in part by translocation of amphibians via global commercial trade (Schloegel et al., 2009). Millions of kilograms of amphibians from wild populations are transported globally each year via the pet and food trades (Schlaepfer, Hoover, & Dodd, 2005). *Bd* may have been recently introduced around the world via trade (James et al., 2009; Morgan et al., 2007; Rachowicz et al., 2005) leading to its current distribution. While *Bd* is widespread in its occurrence, not all areas that are positive for *Bd* have been associated with population declines (Berger et al., 1998; Olson et al., 2013; Ouellet, Mikaelian, Pauli, Rodrigue, & Green, 2005). For instance, Bd is pervasive throughout North America (Olson et al., 2013; Ouellet et al., 2005), yet related declines have only been noted in the western United States (Muths, Corn, Pessier, & Green, 2003). Isolates from different locations vary genetically (Fisher et al., 2009), which may result in differences in host responses (Berger, Marantelli, Skerratt, & Speare, 2005; Farrer et al., 2011; Retallick & Miera, 2007). Potentially, the impacts of *Bd* on hosts could vary by location with more virulent strains associated with areas experiencing declines.

Responses of hosts may also vary with environmental factors that influence host body condition. Immune defenses in response to pathogens are costly to build, maintain, and deploy; therefore, trade‐offs may exist between mounting immune responses and other energetic activities such as growth, reproduction, and thermoregulation (Lochmiller & Deerenberg, 2000). Hosts with poor body condition may be less able to mount effective immune responses because these energetic trade‐offs may be more pronounced. Therefore, host size, as a measure of host condition, may predict outcomes of pathogen exposures. In amphibians, conditions in the larval environment, such as density of conspecifics, can determine size at metamorphosis; larger amphibian body size is associated with increased overwinter survival, earlier time to first reproduction, and increased fecundity (Berven, 1990; Earl & Whiteman, 2015; Semlitsch & Wilbur, 1988). For some amphibian species, the size of individuals has been linked to higher likelihood of *Bd* infection (Kriger, Pereoglou, & Hero, 2007; Searle et al., 2011) and survival after infection (Carey et al., 2006; Garner et al., 2009). Hosts of larger sizes may be more likely to be infected because of their increased ability to mount immune responses and survive. Understanding the links between virulence across isolates and variation in host responses across environmental conditions is critical to understanding how the movement of hosts and their associated pathogens influences distributions of infectious diseases.

Here, we examine the effects of different isolates of *Bd*, two from areas experiencing amphibian declines (El Copé, Panama, and California, USA) and two from areas without declines (Ohio and Maine, USA), on the terrestrial growth and survival of American toad (*Anaxyrus americanus*) metamorphs reared at low or high densities during the larval stage, which resulted “in a relative difference” in body condition. We chose American toad metamorphs in this study because they are sensitive to *Bd* in the laboratory (Gahl, Longcore, & Houlahan, 2012; Wise, Rumschlag, & Boone, 2014) and are infected with Bd in natural populations (Longcore, Longcore, Pessier, & Halteman, 2007; Richards‐Hrdlicka, Richardson, & Mohabir, 2013), which makes this species an ideal model organism for assessing variation in *Bd* isolates’ virulence across environmental conditions. We predicted that isolates from California and Panama would be more virulent than Ohio and Maine, having stronger effects on host growth and survival. Across *Bd* isolates, smaller toads from high‐density larval environments would suffer greater effects on host growth and survival compared to toads from low‐density larval environments.

## 2 MATERIALS AND METHODS

### 2.1 Animal care and collection

Seventeen partial American toad (*Anaxyrus americanus*) egg masses were collected on 12 and 18 April 2014 from Rush Run Wildlife Area (Preble County, Somerville, OH, USA). Eggs were hatched and held in the laboratory at 17 C with a 12‐hr:12‐hr light:dark cycle until tadpoles reached free‐swimming stage (Gosner stage 25 [Gosner, 1960]). We fed tadpoles powdered tropical fish flakes (TetraMin, Tetra Holding) ad libitum until 22 April 2014 when we move them to outdoor mesocosms at Miami University's Ecology Research Center (Oxford, OH, USA). Outdoor 1,000 L mesocosms contained 1 kg leaf litter, 1,000 L water, algae and zooplankton inoculates, and American toad tadpoles at either low density (30 tadpoles per mesocosm) or high density (90 tadpoles per mesocosm). Larval density was manipulated to generate two size classes of metamorphs. We reared tadpoles in mesocosms through metamorphosis. After metamorphosis, they were transferred to the laboratory where individual toads were weighed and held in holding terraria (~38 L capacity; 62 cm × 27 cm × 32 cm) at a density of ~80 toads per terraria. Toads were held in holding terraria for ≤25 days until the start of the experiment when they were transferred into experimental terraria (see below). Terraria contained layers of 1.5 cm pea gravel, 2.5 cm topsoil, and water dishes. Toads were group fed three 3.8‐mm crickets per toad three times weekly.

### 2.2 Experimental Design

We examined the effects of larval density (30 tadpoles/1,000 L [low] or 90 tadpoles/1,000 L [high]) and *Bd* exposure of varying isolates (absent; Sierra Nevada, USA [JEL 213]; El Copé, Panama [JEL 423]; Maine, USA [JEL 404]; Ohio, USA [JSOH 01]) with five replicates of each treatment, for a total of 50 experimental units in the terrestrial phase. An experimental unit was a group of eight toads housed in a single terrarium. Two isolates were from areas with records of *Bd*‐associated amphibian population declines (Sierra Nevada, USA [JEL 213]; El Copé, Panama [JEL 423]), and two were from areas without noted *Bd*‐associated declines (Maine, USA [JEL 404]; Ohio, USA [JSOH 01]), which were acquired from Joyce Longcore (University of Maine, USA). Treatments were randomly assigned to terraria (34 cm × 22 cm × 27 cm) containing groups of eight toads, a water dish, an upturned dish for refugia, and 1.5 cm of pea gravel covered by 2.5 cm of topsoil. Terraria were held at 22 °C on a 12‐hr:12‐hr light:dark cycle.

On 22 June 2014 (experimental day 0), we weighed individuals and gave each a toe clip for identification within the terrarium; two toes or less were cut from each toad. To prevent infection and relieve pain, cut toes were treated with an antiseptic and analgesic ointment, Bactine (Bayer HealthCare LLC). We sprayed terraria everyday with dechlorinated water to keep soil moist and changed water in water dishes each week. We fed groups of toads calcium‐dusted crickets three times per week. Crickets increased in size (from 3.8 to 6.4 mm) and number (from three to 12 crickets/toad/feeding) throughout the time of the experiment.

On 23 June 2014 (experimental day 1), individuals were exposed to *Bd* treatments for 12 hr. Each toad was exposed in a ventilated plastic Petri dish containing 8 ml dechlorinated water and 1 ml of a *Bd* treatment solution (see below). After the exposure, toads were returned to the terraria. We cultured *Bd* on 1% tryptone agar plates using standard protocols (Longcore, Pessier, & Nichols, 1999). To generate *Bd* isolate treatment solutions, *Bd* zoospores were harvested by flooding each *Bd* plate with 4 ml dechlorinated water. For *Bd*‐absent treatments, we added 4 ml of water to plates without *Bd* cultures. We collected zoospores from the plates after 30 min. We calculated the concentration of zoospores in each isolate stock solution using a hemocytometer and diluted stocks with greater zoospore density until all stocks contained 3 × 10^6^ zoospores/ml. Individual metamorphs in *Bd*‐present treatments were exposed to 3.33 × 10^5^ zoospores/ml.

Survival and growth of toads were measured for 73 days in the terrestrial environment. The length of the duration of the experiment, comparable to Wise et al. (2014), was chosen to assess the long‐term effects of *Bd* exposure and larval density on hosts. We monitored survival each day and weighed individual toads weekly to measure growth. To confirm infection by *Bd* from toads, we made wet‐mount slides of epidermal skin sloughs and examined skin samples using a compound light microscope (100×–400× magnification) for *Bd* zoosporangia for toads that died from experimental day 1 until experimental day 40 (Gahl, Pauli, & Houlahan, 2011). Surviving individuals were euthanized by chemical and physical means. Frogs were exposed to a 1% solution of MS222 (tricane methane sulfonate) buffered with sodium bicarbonate until the cessation of movement. Then, they were double pithed, destroying both the spinal cord and the brain. Protocols for this experiment were approved by Miami University's IACUC under protocol number 827.

### 2.3 Statistical analysis

We tested for the effect of *Bd* exposure, larval density (i.e., competition), and their interaction on American toad metamorph survival over 73 days using a generalized linear mixed model with a binomial distribution and a logit link function. Terrarium was designated as a random factor. We tested for the effect of *Bd* exposure, larval density, and their interaction on American toad initial mass on experimental day 0, the number of days that toads survived, change in mass (mass on experimental day 66—mass on experimental day 0), and growth over 66 days using mixed linear models with Gaussian distributions and terraria designated as a random factor. The last day all animals were weighed was day 66. To improve the model fit, we log‐transformed initial mass on experimental day 0, days survived, and masses when analyzing growth over 66 days. To test for treatment differences, we performed multiple‐comparisons tests of the least‐squared means of 73 days survival and log‐transformed number of days toads survived and change in mass. All analyses were completed with SAS 9.4 using the PROC GLIMMIX (73 days survival) procedure, or the PROC MIXED procedure (days survived, change in mass, growth over 66 days) (SAS Institute). Type three tests of fixed effects were evaluated for generalized linear mixed models, and type III sums of squares were evaluated for linear mixed models.

## 3 RESULTS

We measured infection in toads that died using light microscopy of skin samples. Five *Bd* exposed metamorphs died without observable *Bd* zoosporangia: three on experimental day 1 during *Bd* exposures, one on experimental day 2, and one on experimental day 7. All other exposed toads that died during the experiment that were examined had *Bd* zoosporangia visible with light microscopy. Deceased toads in control treatments that were not exposed to *Bd* did not show signs of infection.

**Figure 1:**
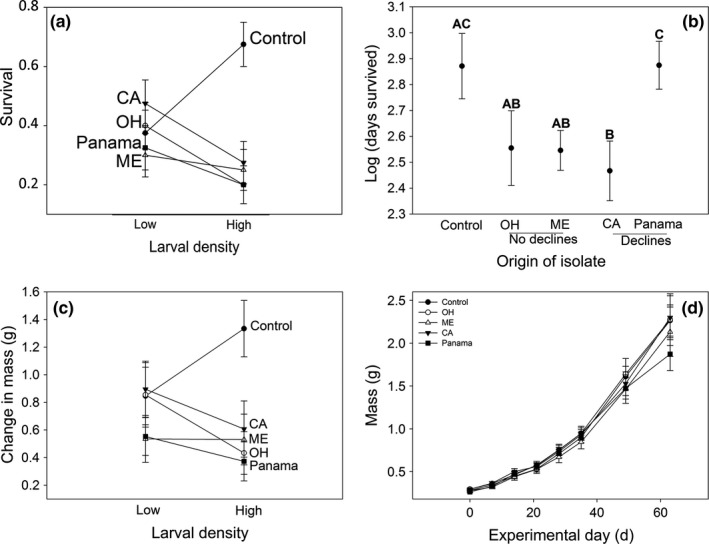
(a) Survival of American toad metamorphs that were reared at different larval densities (low, 30 tadpoles/1,000 L; high, 90 tadpoles/1,000 L) and, as metamorphs, were exposed to *Bd* isolated from areas associated with amphibian declines (CA, Panama), *Bd* isolated from areas not associated with amphibian declines (OH, ME), or American toads not exposed to *Bd* (control). (b) Days survived for American toad metamorphs that were exposed to *Bd* isolates or the control and then were reared for 73 days in the terrestrial environment. Shared letters indicate no significant difference in pairwise comparison. (c) Change in mass for American toads that were reared at different larval densities and, as metamorphs, were exposed to *Bd* isolates or the control. (d) Terrestrial growth over 66 days of American toad metamorphs that were exposed to *Bd* isolates or the control. Metamorphs were reared for 73 days in the terrestrial environment. Mean ± 1 *SE* are shown

Terrestrial survival of American toads was impacted by *Bd* and the interaction of *Bd* and larval density (Table 1), but not by larval density alone. Survival was greatest with larval high‐density and no *Bd* exposure compared to any other treatment, including the larval low‐density treatments (Figure 1a). When exposed to *Bd*, survival in the terrestrial stage was reduced for metamorphs from high‐density larval mesocosms relative to metamorphs from low‐density larval mesocosms (Figure 1a), but the magnitude of this reduction in survival across larval density varied with isolate. For instance, across larval density treatments, survival of toads from the high‐density mesocosms was reduced more for metamorphs exposed to isolates from California, Ohio, and Panama compared to metamorphs exposed to the Maine isolate (Figure 1a).

**Table 1:** Summary of linear mixed model analysis of treatments and their interaction on survival, time of death, change in mass, and growth over the course of the study of American toad metamorphs

Response	Source of variation	*df*	*F*	*p*
73‐d survival	Density	1, 350	1.87	.1726
	*Bd*	4, 350	3.94	**.0039**
	*Bd* × density	4, 350	3.61	**.0067**
Days survived	Density	1, 211	0.00	.9584
	*Bd*	4, 211	2.66	**.0337**
	*Bd* × density	4, 211	0.21	.9334
Change in mass	Density	1, 350	0.49	.4850
	*Bd*	4, 350	4.49	**.0015**
	*Bd* × density	4, 350	3.43	**.0090**
Growth	Density	1, 1,050	1.43	.2320
(Between Subjects)	*Bd*	4, 1,050	0.26	.9022
	*Bd* × density	4, 1,050	0.96	.4284
(Within Subjects)	Time	7, 1,050	367.98	**<.0001**
	Density × time	7, 1,050	0.42	.8877
	*Bd* × time	28, 1,050	1.54	**.0369**
	*Bd* × density × time	28, 1,050	1.23	.1939

*p* values <.05 are bolded.

The number of days that toads survived was impacted by the isolate of *Bd* exposed to, but not by larval density or the interaction of these treatments (Table 1). Compared to the control, metamorphs died more quickly when exposed to the *Bd* isolate from California but not when exposed to *Bd* isolates from Ohio, Maine, or Panama (Figure 1b).

Change in mass of American toads was impacted by *Bd* and the interaction of *Bd* and larval density (Table 1), but not by larval density alone. The change in mass over the course of the experiment was greatest in with larval high‐density and no *Bd* exposure compared to any other treatment, including the larval low‐density treatment (Figure 1c). When exposed to *Bd*, change in mass in the terrestrial stage was reduced for metamorphs from high‐density larval mesocosms relative to metamorphs from low‐density larval mesocosms (Figure 1c), but the magnitude of this reduction across larval density varied with isolate. For instance, across larval density treatments, change in mass was reduced more for metamorphs exposed to isolates from California, Ohio, and Panama compared to metamorphs exposed to the Maine isolate (Figure 1c). Examining growth over time, *Bd*, but not density or the interaction of density and *Bd*, impacted the growth of American toad metamorphs (Table 1, Figure 1d). At the start of the study, American toad metamorphs from low larval density treatments were 11% larger than toads from high larval density treatment (low larval density treatment: 0.253 ± 0.0146 g, high larval density treatment: 0.227 ± 0.0145 g [least squares mean ± *SE*]; *F*_1, 350_ = 10.38, *p *=* *.0014). However, toad size did not differ significantly by *Bd* treatments (*F*_4, 350_ = 1.71, *p *=* *.1464) or the interaction of density and *Bd* (*F*_4,350_ = 1.31, *p *=* *.2652). Growth of American toad metamorphs over time was reduced with exposure to the *Bd* isolate from Panama and Maine compared to exposure with *Bd* isolates from Ohio, California, and the *Bd* control (Figure 1d).

## 4 DISCUSSION

The world is evermore connected thanks to globalization, which has lead to increased translocation of hosts and their pathogens. This movement results in novel pairings of pathogen strains and naïve hosts. Evaluating variation in host responses to pathogen strains across varying environmental conditions is essential to understanding how the translocation of organisms influences the outcomes of novel pathogen and host pairings in natural environments. In natural populations, an amphibian chytrid fungus *Bd* is widespread in its occurrence, but the presence of *Bd* is not always associated with amphibian declines (Daszak et al., 2005; Olson et al., 2013; Ouellet et al., 2005). Novel pairings of hosts and pathogens could in part explain outbreaks of chytridiomycosis that have resulted in mass mortality events. Outcomes of these novel pairings may vary with environmental conditions that influence host condition, which is linked to the ability of hosts to respond to pathogens. Our study evaluated whether responses of a vulnerable host, reared in high and low larval conditions, which influenced host size, would vary with exposure to *Bd* isolates from locations associated with the presence and absence of amphibian population declines. We predicted that exposure to *Bd* isolates from areas where amphibian population declines have occurred, California and Panama, would result in greater negative effects than isolates from areas where population declines related to disease have not been observed, Ohio and Maine, and that these negative effects of *Bd* would be stronger for hosts reared in high larval density conditions compared to low larval density. Our results provide a partial explanation for the pattern that exists between *Bd* occurrence and amphibian declines by demonstrating that host responses to *Bd* can vary with isolate and larval environment, which influences host body condition.

Our results indicate that variation in host responses to *Bd* isolates exists, but does not vary based on whether or not isolates are associated with locations of amphibian population declines, pointing to the need for caution against introductions of novel strains. Local selection pressures may drive these observed variations in the effects of isolates on host growth and survival, even though isolates from areas of decline did not impact American toads more strongly than isolates from areas where declines are absent. For instance, the effects of the local Ohio isolate did not impact hosts less than any of the other isolates, including those from areas associated with declines. The geographic range of *Bd* is large, encompassing locations of diverse environments (Olson et al., 2013). Local adaptations to these environments over different time scales may drive the observed differences in responses of hosts in the current study. However, our results suggest that amphibian declines related to *Bd* in parts of the world may not be solely caused by variation in isolate virulence. While genetic variation in isolates could contribute to declines in some areas under some conditions, likely variation in host responses to other factors within the environment that influences host susceptibility is important for determining the outcome of pathogen exposures on hosts.

We found no clear pattern with isolate origin and virulence, suggesting that effects of pathogens based on their locations of origin are unpredictable in this system. Movement of a pathogen to a new location may pose risks to hosts at the new location regardless of its place of origination and effects on hosts native to the place of origination. Characterizing isolate virulence by origin has limited usefulness and isolates of chytrid fungi need not be novel to potentially threaten amphibian populations, even in localities without previously noted declines. We found that the native Ohio isolate caused negative effects on hosts similar to isolates from areas associated with amphibian population declines. We reiterate the call to limit human‐aided host and pathogen movement through trade and translocation. Limitation of the movement of hosts will prevent novel pairings of pathogens and hosts, which could result in unanticipated consequences.

Perhaps most interestingly, our results also show that increased competition in the larval stage, which affects size at metamorphosis, may increase the impact of *Bd*. Across isolates, survival and growth of smaller hosts reared as larvae at high densities were generally reduced in response to *Bd* exposure, indicating that host condition can determine the outcome of pathogen exposures on hosts. The effect of density can vary with the identity of the isolate, indicating that under certain conditions exposure to high‐density larval environments can increase the effect of some isolates. The conditions that hosts experience may induce or increase susceptibility; and the magnitude of this effect varies according to which isolate hosts are exposed. These results support that host condition is an important factor that in part determines susceptibility to disease (Lochmiller & Deerenberg, 2000; Searle et al., 2011; Wilcoxen, Boughton, & Schoech, 2010). The mechanism of this observed effect might be linked to host size and energetic abilities to defend against pathogens. Physiological trade‐offs might exist that lead to hosts of higher body condition as more likely to grow and survive when exposed to *Bd* (Lochmiller & Deerenberg, 2000). Other studies in different systems support that larger hosts can have more developed immune systems (Møller, Christe, Erritzoe, & Mavarez, 1998; Wilcoxen et al., 2010). The larval environment may be critical for determining the ability of hosts later in life to combat infectious diseases; quality larval environments with ample space and resources may decrease susceptibility to *Bd*, while conditions that increase competition for food resources, such as drying ponds or increased density of hosts during breeding may increase risk.

The effects of *Bd* on populations of amphibians in the Midwestern United States for species like the American toad are not well understood because research in this field generally focuses on areas in which widespread mortality events have occurred, such as Central and South America and Australia. While no reported mortality events from *Bd* have been reported in the Midwestern United States, our research suggests that amphibian hosts may still be at risk to *Bd,* especially if conditions impact host condition and increase susceptibility. Our results support the limitation of movement of hosts and pathogens to new environments because of the unpredictability of outcomes of novel pairings, and conservation of high‐quality aquatic and terrestrial habitat for amphibian hosts in populations that may be at risk of outbreaks of chytridiomycosis to decrease the strength of effects on hosts. Consideration must be given to variation in host responses across strains and environmental conditions if our goal is to limit effects of pathogens on host populations.

## CONFLICT OF INTEREST

The authors declare no conflict of interests.

## AUTHOR CONTRIBUTIONS

SLR and MDB conceived the study design. AKB conducted the experiment under the guidance of SLR and MDB. MDB and SLR analyzed the data. AKB, SLR, and MDB interpreted the results. AKB and SLR wrote the manuscript, and MDB provided editorial advice.

## DATA ACCESSIBILITY

The dataset will be made available in Dryad upon publication.
